# Additive-Free Method for Enhancing the Volume Phase Transition Rate in Light-Responsive Hydrogels: A Study of Micro-Nano Bubble Water on PNIPAM-co-AAc Hydrogels

**DOI:** 10.3390/gels9110880

**Published:** 2023-11-07

**Authors:** Saho Kuroki, Masaya Kubota, Ryota Haraguchi, Yushi Oishi, Takayuki Narita

**Affiliations:** Department of Chemistry and Applied Chemistry, Saga University, 1 Honjo, Saga 840-8502, Japan

**Keywords:** volume phase transition, micro-nano bubble water, photo-thermal conversion, shrinking behavior

## Abstract

Light-responsive hydrogels containing light-thermal convertible pigments have received interest for their possible applications in light-responsive shutters, valves, drug delivery systems, etc. However, their utility is limited by the slow response time. In this study, we investigated the use of micro-nano bubble water as a preparation solvent to accelerate the volume phase transition kinetics of poly(N-isopropylacrylamide-co-acrylic acid) (PNIPAM-co-AAc) hydrogels. The hydrogels were characterized by dynamic light scattering (DLS) and dissolved oxygen (DO) measurements. The mechanical properties, surface morphology, and chemical composition of the hydrogels were analyzed by Young’s modulus measurements, scanning electron microscopy (SEM), and Fourier transform infrared (FT-IR) spectroscopy, respectively. The results showed that hydrogels prepared with bubble water changed the volume transition rate by more than two orders of magnitude by simply changing the standing time of the bubble water for only a few hours. The cooperative diffusion coefficients obtained from the light-induced volume transition kinetics correlated linearly with Young’s modulus and metastable state swelling ratio. Our results suggest that bubbles act as efficient water channels, thereby modulating the response rate and providing a simple, additive-free method for preparing hydrogels with a wide range of response rates.

## 1. Introduction

Thermo-responsive hydrogels containing pigments exhibit a light-induced responsive volume phase transition based on the light-to-heat conversion of the dye additives [1,2,3,4,5,6,7]. This material has a simpler and more feasible light-thermal conversion-based light response process than other light-responsive materials [8,9,10]. Light-responsive hydrogels are expected to find applications as shutters [11], valves [12,13], and drug carriers in drug delivery [14,15,16]. When considering such applications, precise control of the stimulus-response rate is critical. This rate control mechanism would provide notable advantages in treating medical conditions requiring prolonged drug administration [17,18] or precise dosing regimens [19,20]. The precise regulation of drug delivery can enable control over the timing and administration of dosage. Quick response to the environmental response is essential when controlling the system’s movement. However, the typical macroscale hydrogel shrinkage rate can take several days to reach equilibrium shrinkage [21,22]. As a result, macroscale hydrogels exhibit significantly slower response rates than other light-responsive materials. These slow dynamics of hydrogels can be attributed to forming a condensed layer, commonly called a skin layer, on the hydrogel surface during the shrinkage process [21,22,23,24,25,26]. The skin layer consists of an aggregated, high-density polymer network, and the narrower mesh size causes an increase in the area of frictional resistance between the polymer and water, severely restricting water flow within the hydrogel network, especially drainage. Several approaches have been investigated to address the slow stimulus response issue [27,28,29,30,31,32,33,34]. Previous studies have found that incorporating surfactants [28,31] and dangling chains into the hydrogel [29,30] is useful in overcoming the slow response phenomenon.

However, current methods have drawbacks related to the elimination of additives and the complexity of their preparation. In the above solution, forming a porous structure inside the gel prevents the development of the dense layer and allows water to drain quickly from the interior of the gel.

Micro or nano bubble water is a liquid medium characterized by tiny bubbles evenly distributed throughout the water [35,36,37,38,39]. The bubbles are remarkably small, with diameters ranging from tens to hundreds of nanometers. These bubbles are stable in the water medium and remain dispersed for an extended period. Hydrogels formed using micro-nano bubble water (Hereafter in this manuscript, ‘micro-nano bubble water’ will be referred to simply as ‘bubble water’) have the potential to form a porous structure because hydrogels will not be polymerized in the bubbles. The microporous structure of the water pathway [29,34], which allows water to pass through the gel easily, would prevent the formation of the skin layer and provide a faster and smoother stimulation response. Other advantages of using bubbles as templates for the porous structure are that adding reagents is unnecessary in the post-treatment process, and the preparation process is straightforward. Rapidly responsive hydrogels could be active in designing new hemostatic agents [40] and drug delivery systems [41]. In this context, the porous structure enabled by bubble water could provide a versatile platform for such responsiveness [42]. This study aims to investigate the impact of bubble water on the volume phase transition behavior.

In this study, we investigated how bubble water in the gel preparation affects light-induced volume changes using its rapid heat transfer via light-thermal conversion. This approach overcomes the difficulty of changing the gel temperature solely by controlling the water medium. For this purpose, thin slices of PNIPAM-co-AAc hydrogel containing photothermal pigments were used as samples. The disks were irradiated under visible light, and the volume change behavior was evaluated by calculating the characteristic time τ and the specific swelling ratio obtained from the gel shrinkage based on the area change behavior of the disk gel. The size of bubbles in bubble water depends on the standing time. Therefore, the influence of water standing time on the gel structure and the transition behavior were investigated. SEM was used to study the impact of bubble water on the gel structure. The chemical composition of the gels prepared with bubble water and its effect on the transition was confirmed by FT-IR for the dried samples and by the temperature-volume phase diagram.

The principal innovation we focus on here is introducing a pioneering additive-free method to expedite the volume change rate in light-responsive gels. Our results show that using bubble water as a solvent for preparing light-induced volume transition gels accelerates the transition rate approximately 100 times faster than gels without bubble water. The mechanism of this acceleration is discussed through structural and physical property measurements.

## 2. Results and Discussion

### 2.1. Properties of Bubble Water and the Effect of Standing Time on Its Properties

First, to evaluate the properties of the bubble water used as a solvent for hydrogel preparation, the bubble size and oxygen concentration of the bubble water were studied using DLS measurements and DO concentrations. Since oxygen in water is known to be a radical scavenger, the oxygen concentration would affect the formation of polymer networks.

#### 2.1.1. DLS Measurements

**Figure 1:**
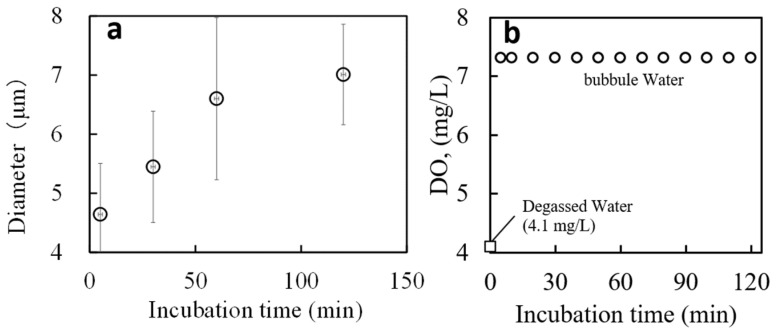
Dependence of (**a**) bubble size and (**b**) dissolved oxygen concentration on the incubation time after bubbled water preparation. Average hydrodynamic bubble size as determined by DLS measurements, plotted against incubation time, which represents the duration each water sample was exposed to the atmosphere post-generation.

Figure 1a shows the bubble size in the bubble waters measured by the DLS measurement plotted against the incubation time after the bubbles were generated. The average bubble size increased from 4.5 µm to 7 µm over 2 h. The stability of the bubbles is maintained due to the solubility effect and their high internal pressure [43]. Various factors, including generation methods, measurement techniques, and the purity, temperature, and pressure of the water used, influence the lifetime and stability of the micro-nano bubbles. Consequently, it is challenging to provide a general duration for their nanosized persistence in pure water [35,43]. For a single bubble with an initial radius of 200 nm, the estimated lifetime ranges from 10 to 100 s. Bubbles bigger than nanometers tend to have shorter lifetimes than micro-nano bubbles in pure water [44,45,46,47]. This is due to the pressure acting on the surface of the bubble and the tendency of the gas inside the bubble to dissolve in water. Since the bubble water used in our experiment was prepared from pure water and was relatively large and micronized, coarsening is expected to have progressed within this time frame.

#### 2.1.2. DO Concentration

DO concentrations were 4.1 mg/L for degassed water and 7.3 mg/L for bubble water and remained stable within measurement error for 120 min after preparation (see Figure 1b). The bubble water had approximately double the oxygen concentration.

### 2.2. Effect of Bubble Water Used in the Preparation on the Static Properties of the Gel

#### 2.2.1. Mechanical Strength Measurement

**Figure 2:**
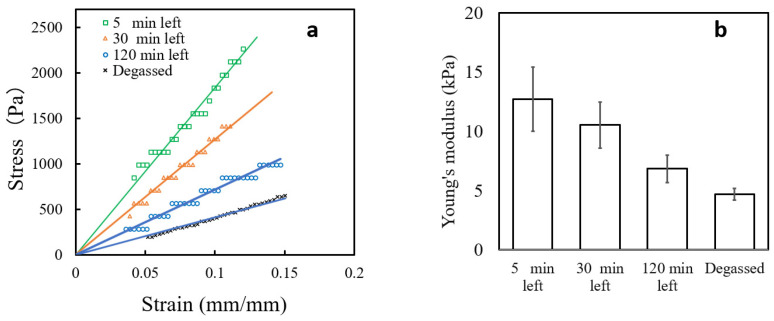
Mechanical properties: (**a**) stress–strain curves and (**b**) Young’s modulus of PNIPAM-co-AAc hydrogels prepared using the degassed water or the bubbled waters left for given periods. Young’s modulus calculations were based on the slope of the linear portion of the curve in Figure 2a.

Figure 2a shows the linear sections of the stress–strain curves of gels prepared with degassed water or bubbled water left for different aging times. The gel obtained from degassed water has the lowest Young’s modulus at 4.7 kPa, making it the softest sample. Conversely, the gel obtained from 5-min aged bubble water has the highest Young’s modulus at 12.7 kPa. These results indicate that bubble water significantly affects the mechanical properties of the gels. Gels made with bubble water with longer aging times are softer, which could be related to the size of the bubbles, and will be discussed in Section 2.3.

Interestingly, gels synthesized with degassed distilled water exhibited the lowest average Young’s modulus at 4.7 kPa. These findings imply that micro bubbles and nano bubbles contribute to increased gel stiffness. The mechanical properties of gels differ significantly between degassed and bubble water, affecting the kinetics and control of polymerization reactions [48,49]. While oxygen can serve as a free radical initiator [50,51], it may also quench radicals, inhibiting polymerization [52,53]. However, this oxygen-radical interplay insufficiently accounts for the observed variations in the elastic modulus due to effervescent water aging. Given that the bubble concentration remains saturated for up to 180 min, the bubble size likely influences the network architecture, affecting the modulus [54]. Smaller bubbles may enhance network homogeneity by uniform monomer dispersion or equalizing cross-link distances. Further investigations to substantiate these hypotheses are warranted but fall outside the scope of this report.

#### 2.2.2. SEM Analysis

**Figure 3:**
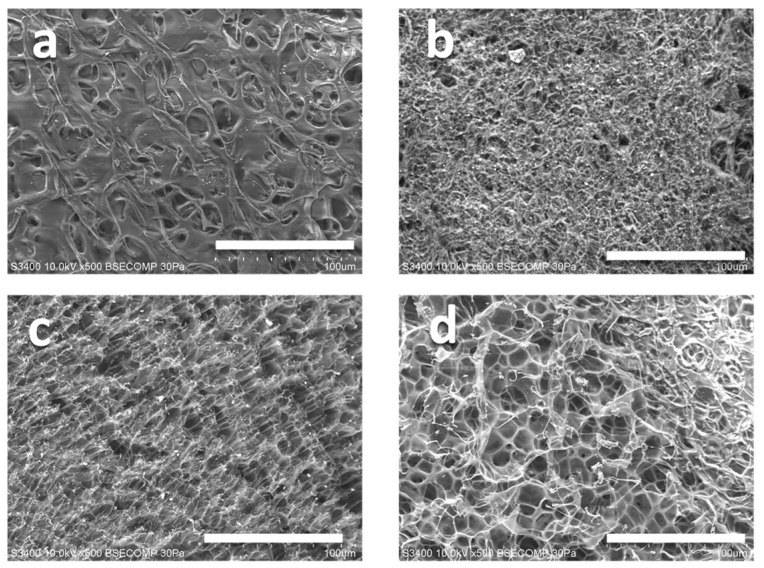
SEM images of the aerogel surfaces freeze-dried PNIPAM-co-AAc hydrogels prepared using (**a**) degassed water, bubbled water left for (**b**) 5 min, (**c**) 30 min, and (**d**) 120 min. The scale bars in the micrographs represent 100 µm.

Figure 3 shows SEM images illustrating the surface morphology of aerogels prepared using bubble water as a solvent followed by freeze-drying. A comparative analysis between aerogels synthesized with bubble water and those using degassed water reveals distinct differences in surface topography. In particular, aerogels formed with bubble water exhibit a significantly rougher surface characterized by a sponge-like morphology. This textured surface is commonly observed in aerogels prepared by freeze-drying hydrogels and is attributed to the formation of ice nuclei during the freezing phase of the hydrogel [55,56]. The intricate dimensions and characteristics of the surface textures are closely related to the parameters of the freezing and freeze-drying processes employed. While SEM analysis revealed a correlation between increased pore size and the aging time of bubble water, definitive evidence linking these features to the residual bubbles is lacking. We postulate that cavities and voids within the matrix may act as central ice nucleation sites during the freezing process, thereby influencing the surface morphology of the aerogel. SEM analysis suggests that bubble formation in the solvent during the polymerization phase induces significant changes in the microstructure of the resulting gel. The apparent porous architecture shown in this figure plays a critical role in facilitating the expulsion and subsequent absorption of water necessary for gel deformation. In particular, this has implications for the deformation rate during volume transitions. However, additional studies are required to understand the mechanistic basis fully.

#### 2.2.3. FT-IR Spectra

**Figure 4:**
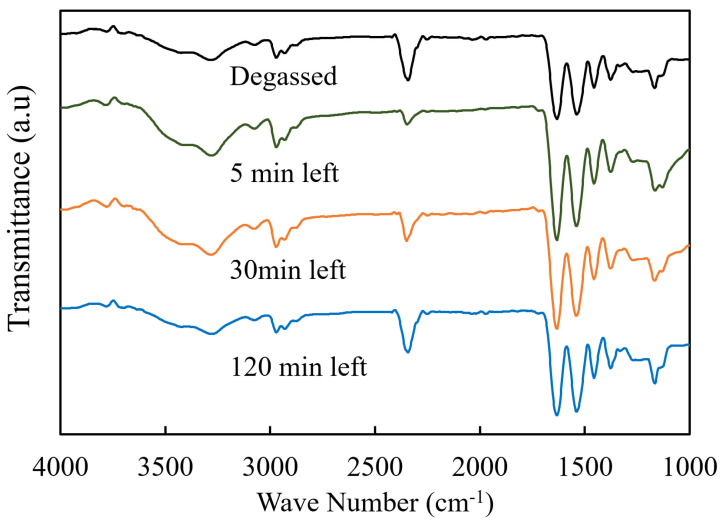
FT-IR spectra of dried PNIPAM-co-AAc hydrogels prepared using bubbled water left for specified times (in minutes) and degassed water. The intensities of the spectra have been adjusted to facilitate the inclusion of all spectra in this figure.

Figure 4 shows the FT-IR spectra of dried PNIPAM-co-AAc gels prepared with bubbled water left for the given minute and with degassed water. Unique absorption bands were observed in all samples analyzed at about 1650 cm^−1^ and 1530 cm^−1^. These bands are attributed to the C=O stretching vibrations inherent in amide linkages and the N-H bending vibrations, respectively. Such spectral features confirm the successful polymerization of PNIPAM and the formation of amide bonds. In addition, an absorption peak near 1700 cm^−1^ can be attributed to PNIPAM and the C=O stretching vibrations of the co-polymerized AAc monomer. This comparative spectral analysis concludes that the presence or absence of bubbles during gelation has little impact on the chemical composition of gels.

#### 2.2.4. Temperature-Swelling Ratio Phase Diagram

**Figure 5:**
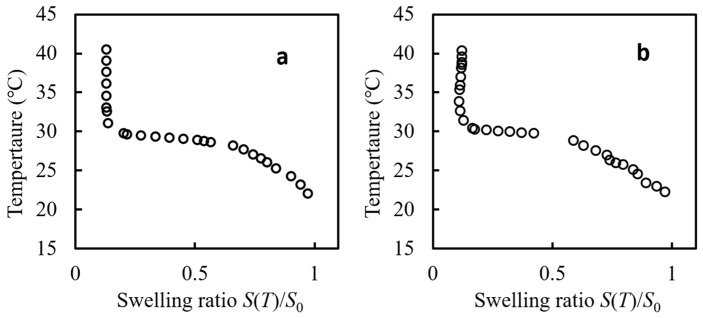
Swelling ratio *S*(*T*)/*S*_0_ of PNIPAm-co-AAm hydrogels plotted as a function of temperature. Hydrogels were synthesized using (**a**) degassed water and (**b**) bubble water left for 120 min.

To elucidate the role of bubble water on the thermodynamic properties of the hydrogels, we carefully analyzed the swelling ratio at temperatures close to the phase transition. In both the volume–temperature phase diagrams, as shown in Figure 5a,b, of gels prepared with the bubble water and with degassed water, the gels showed a transition temperature of 28.5 °C during both processes of heating and cooling, and the corresponding curves confirmed the consistency: the transition temperature of the PNIPAM-co-AAc gel was 4–5 °C lower than that of a typical PNIPAM gel prepared with NIPAM alone. This decrease in the transition temperature would primarily be due to adding 0.1% AAc [57]. This difference is in agreement with previously reported studies. Notably, the transition temperatures for both gels were identical, and the difference in shrinkage at equal weights was minimal. This supports the conclusion of the result of FT-IR that variables such as the presence of bubbles and variations in oxygen levels have little or no effect on the chemical composition of the prepared gels. We assert that the observed phase diagrams are largely congruent; however, a discernible discrepancy manifests predominantly in proximity to the phase transition point. The phase diagram was acquired via a 0.02 K/min temperature ramping rate. Considering the limitation that thermal equilibrium may not have been fully attained due to the continuous temperature increment is imperative. This lack of complete equilibration is likely attributable to the phenomenon of critical slowing down [26,58,59], which is characterized by significantly retarded kinetics near the phase transition point.

### 2.3. Effect of Bubble Water Used in the Preparation on the Dynamic Properties of the Gel

#### Light-Induced Volume Phase Transition Behavior

**Figure 6:**
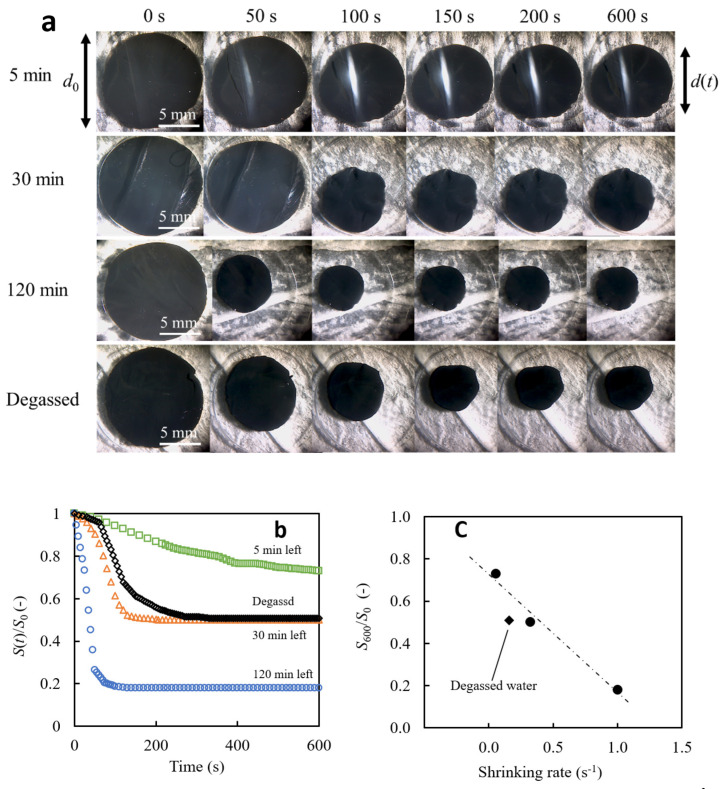
Morphological change in PNIPAM-co-AAc hydrogels after LED irradiation, prepared with bubbled water left standing for specified times after nano-micro bubble generation; (**a**) Time-lapse photographs captured from a top view, illustrating gels prepared with bubble water left to stand for 5, 30, and 120 min; (**b**) time course of the swelling ratio for these samples; (**c**) correlation between the maximal shrinking rate and the swelling ratio in the metastable state *S*_600_/*S*_0_ (•).

To investigate the kinetics of the volume changes, we monitored the swollen state of the gels before and after LED light irradiation. Figure 6a is a series of photographs of gel disks captured at given time points to investigate the size change behavior of PNIPAM-co-AAc gel disks prepared using bubble water and degassed water under light irradiation (see Videos S1–S4). A comparison of these sequential photographs shows that the leaving time of the bubble water affects the gel shrinking rate. From the series of images, simultaneous comparisons after light irradiation reveal distinct kinetic behaviors of the gels. Specifically, the gel prepared with a standing time of 5 min exhibited the highest extended shrinkage rate. Conversely, the gel prepared with water using a standing time of 120 min showed almost complete shrinkage within 100 s. These observations support the hypothesis that gels prepared with water using different bubble water standing times exhibit markedly different kinetic properties. Figure 6b gives the time course of the change in the hydrogel disk’s relative area (area at t = 0 is 1). The gel disk first undergoes a slow shrinkage step, which is then replaced by a rapid shrinkage step, resulting in a metastable stable state (as seen in the typical behavior of gels prepared with degassed water). Upon reaching this metastable state, a significant slowdown in area shrinkage was observed. In other words, the gel undergoes a two-step dynamic to reach the equilibrium swelling value in shrinkage. Most samples reached this metastable state within 600 min after LED irradiation, although as shown in Section 2.2.4. all gels can reach the equilibrium shrinkage state (*S*_∞_/*S*_0_ = 0.12, where the equilibrium gel disk area is in the shrunken and swollen states, respectively) at around 30 °C when the temperature increases over time. The metastable state swelling ratio *S*_600_/*S*_0_ is larger than all samples’ equilibrium swelling ratio in the shrunken state (*S*_∞_/*S*_0_ = 0.12). This means that true equilibrium was not reached by 600 min. The significant slowdown in the metastable state is due to the dense network that forms from the gel surface during shrinkage, and the friction between the water and the polymer network causes a significant delay in the internal water ejection rate [60,61,62]. This figure shows that the water used for gel preparation makes a clear difference in the metastable state swelling ratio (*S*_600_/*S*_0_) and the shrinking rate (d(*S*(*t*)/*S*_0_)/d*t*), which corresponds to the slope of each curve. These values represent the gel disk area at 600 and t seconds after the light irradiation, respectively. Gels prepared with bubble water showed a lower metastable state swelling ratio and a faster rate as time increased. In contrast, hydrogels prepared with 120 min of settled bubble water exhibited the most shrunken metastable state with a significantly accelerated shrinking rate. Notably, as shown in Figure 6c, we can see a linear relationship between the fastest transition rate to this metastable state and the swelling ratio in the metastable state (*S*_600_/*S*_0_) in the metastable state for gels with bubble gel.

Given this relationship, we would not be surprised if there is a relationship between the fast shrinkage rate during the first stage of the early shrinkage process and the average size in water bubbles determined by DLS. Below, we discuss the kinetics underlying the light-thermal response of PNIPAM-co-AAc gels. To derive the relationship between bubble size and gel shrinkage rate, let us consider the diffusion of the gel network during the light response. Here, we briefly review the kinetics associated with the contraction of the gel disk, as reported by Li and Tanaka [63,64,65]. The expansion and contraction behavior of hydrogels theoretically follows the diffusion equation. The interface between the gel and the neighboring solvents causes these phenomena, but the gel does not expand and contract uniformly. In this case, the diffusion motion of solvent molecules is essential, and a cooperative diffusion model can demonstrate gels’ expansion and contraction based on the studies of Tanaka et al. In the case of the expansion and contraction of the gel disk [65,66,67,68], the time-dependent change of its radius *R* of the disk can be expressed at the last stage by the following equation:

(1) $$\ln\frac{u\left(R,t\right)}{u\left(R,0\right)}=-\frac{1}{\tau_{1}}t+\ln B_{1},$$

where *t* represents time, and *u*(*R*,*t*) is the displacement vector of the points in the mesh concerning the final position. $\tau_{1}$ and $B_{1}$ are the contraction relaxation time and a function of the elasticities of the gel, respectively. Thus, from Equation (1), $B_{1}$ and $\tau_{1}$ can be calculated from the logarithmic plot’s intercept and slope. This equation was fitted to each curve in Figure 6b to obtain *τ*_1_ and *B*_1_ for the target gels. For disk-shaped gels, the following specific relationship exists between the relaxation time and the cooperative diffusion coefficient:

(2) $$D_{c}=\frac{l^{2}}{\pi^{2}\tau_{1}}\;,$$

where *l* is the disk gel thickness (here 0.5 mm). The $D_{c}$ obtained from the fitting were 6.1 × 10^−7^, 4.3 × 10^−6^, 2.1 × 10^−5^, and 1.5 × 10^−6^ cm/s for gels obtained in bubble water at 10, 30, and 120 min and degassed water, respectively. The data indicate that changing the settling time of bubble water can modulate the diffusion coefficient in the resulting gels by nearly two orders of magnitude. Furthermore, considering the standard diffusion constants for chemically cross-linked gels (10^−7^ to 10^−8^ cm/s) [65,68], gels synthesized with 180 min aged bubble water can contract approximately two orders of magnitude faster.

**Figure 7:**
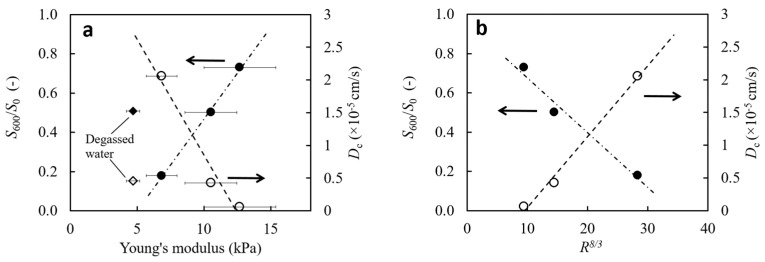
Dependence of the metastable swelling ratio (*S*_600_/*S*_0_) (•) and the network co-diffusion coefficient *D*_c_ (○) on (**a**) the gel’s shear modulus and (**b**) the 8/3 power of the mean bubble radius (*R*^8/3^). Arrows on the plots indicate which y-axis.

The relationship between the obtained cooperative diffusion coefficient and Young’s modulus of the gel and the swelling ratio in the metastable state is shown in Figure 7a. An almost linear relationship between Young’s modulus and the quasi-equilibrium swelling ratio and diffusion coefficient is observed for gels prepared with bubble water. This linear relationship implies that the gel structure, influenced by the presence of bubbles, affects the transition behavior in this region. Young’s modulus of the network is also essentially related to the density of cross-linking points. This linearity also tells us that nano-bubble water can affect the density or distribution of the network formed. The fact that the gel properties of gels prepared with degassed water deviate from this linear relationship suggests that the topology of the vacancies used as water channels for dehydration may differ fundamentally from those prepared with bubble water.

In the most basic model, bubbles provide the primary water channels inside the gel. More giant bubbles create wider water pathways, potentially reducing the friction, thereby accelerating water ejection and decreasing gel elasticity, assuming friction is a dominant factor. Assuming that water is expelled only through these large pores and that the polymer–water friction remains constant, the Hagen–Poiseuille equation becomes relevant. This equation correlates the gel contraction rate d(*S*(*t*)*/S*_0_)/d*t* with the volume of water expelled *Q* as d(*S*(*t*)*/S*_0_)/d*t* = d*Q*^2/3^/d*t*. Consequently, the gel deformation rate is proportional to *R*^8/3^. Figure 7b plots the network diffusion coefficient *D*_c_ against *S*_600_/*S*_0_ as a function of *R*^8/3^ and shows a linear relationship. While further study is required, this correlation supports the hypothesis that bubbles are the primary water conduits in the gel. The observed co-diffusion coefficients up to the skin layer formation suggest that bubble-induced pores are critical in inhibiting the onset of this phase.

A more rigorous comparison can be made using bubble water composed of gasses that do not interfere with polymerization, such as nitrogen, instead of the atmospheric air used in the current study. This improved experimental setup will be the focus of a future report.

## 3. Conclusions

Micropores formed in gels using micro bubbles and nano bubbles as templates can function as channels that facilitate the retention and efflux of water molecules. In other words, these micropores are thought to prevent the formation of a dense layer on the hydrogel surface. Thus, the micropores inside the gel can dramatically shorten and easily regulate the relaxation time. The impact of nano-micro bubble water on the developed gels can be summarized in the following key points: Nano-bubbles significantly modulate Young’s modulus of polymer network structures. There is a direct inverse correlation between bubble size and Young’s modulus of the gel. The gel network diffusion, shrinkage, and water evacuation rate appear to be highly dependent on the micropores in the gel.

The highlight of this article, which is now more clearly articulated, is introducing a pioneering additive-free method for accelerating the volume changing rate in light-responsive gels. This approach fills a critical gap in the current research landscape by providing an innovative solution for improving hydrogel response rates without relying on additives.

## 4. Materials and Methods

### 4.1. Materials

For the synthesis of PNIPAM-co-AAc hydrogels, N-isopropyl acrylamide (NIPAM, 98.0%), acrylic acid (AAc; 98.0%), the crosslinker, N, N’-methylenebis (acrylamide) (MBA, 97.0%), and the initiator, ammonium persulfate (APS, 98%), were obtained from FUJIFILM Wako Pure Chemical Corporation, Osaka, Japan. All chemicals were used without purification.

### 4.2. Preparation of Bubble Water and Degassed Water

The solvents used to prepare PNAIPAM-co-AAc gels were degassed water and water containing high concentrations of bubbles. Degassed water was obtained using ultrapure water (Millipore-Direct Q UV3, Merck) and prepared by stirring the water under a vacuum for about 1 h. The oxygen concentration was reduced to 4.1 mg/L. Bubbles in pure water were prepared using a bubble generator (NB-1, AS ONE Co., Osaka, Japan). The water containing a high concentration of micro-nano bubbles is abbreviated as bubble water. After the bubble water was prepared, the samples were allowed to stand for 5 to 120 min. DO in the bubble water was 7.3 mg/L for at least 120 min after the water was prepared (see Figure 1b).

### 4.3. Preparation of PNIPAM-co-AAc Hydrogel Discs

PNIPAM-co-AAc hydrogels were synthesized by a radical polymerization method. In a 100 mL beaker placed on a magnetic stirrer, NIPAM and AAc were added to 25 mL of the prepared bubble water. The cross-linking agent MBA was introduced and stirred slowly until the solution dissolved. The addition of APS initiated polymerization. The mixed pre-gel solution was filled into the space between parallel plates with a 0.5 mm gap and polymerization was performed in a refrigerator for 24 h at 4 °C. The prepared gels were removed from the mold plate. Then, they were left for 1 day to rinse with deionized water. Using a hollow punch with an inner diameter of 10 mm, the prepared gels were cut into 10 mm circular discs.

### 4.4. Characterization of Bubble Water

#### 4.4.1. DLS

The hydrodynamic size of bubbles in the solvent water used for gel polymerization (degassed water and water with bubbles left for 5, 10, 30, and 120 min) was determined using a particle size analyzer (ELS Z2 Plus; Otsuka Electronics, Osaka, Japan). Each measurement was performed in triplicate to ensure reproducibility. The autocorrelation function obtained from the scattered light intensity was analyzed using the instrument’s built-in software. Particle size distributions were derived by fitting the observed autocorrelation function using CONTIN algorithms, and the reported hydrodynamic diameter represents the intensity-weighted mean diameter.

#### 4.4.2. DO Measurement in Degassed and Bubble Waters

DO concentrations in the aqueous bubbles were measured to clarify differences in the preparation conditions of the synthesized gels. DO concentrations were measured using a PDO-520 (FUSO, Tokyo, Japan). For accurate measurements, the probe was calibrated under atmospheric conditions. Each water sample was gently agitated to ensure uniformity, and the probe was deeply immersed in the sample to obtain stable readings. Measurements were taken every 10 min at 25 °C (from 0 to 10 min, in 5 min intervals) for 120 min to ensure consistency of the readings.

### 4.5. Characterization of PNIPAM-co-AAc Hydrogels

#### 4.5.1. Mechanical Strength Measurement

The elastic modulus of the PNIPAM-co-AAc gel was determined using a RE2-33005B (YAMADEN Co., Ltd., Tokyo, Japan). The experiments were performed under the following conditions: Measurement strain rate: 30%, measuring speed: 1 mm/sec, contact area diameter: 3 mm, fixture used: Φ3 mm, application temperature: 24 °C. Twelve samples of each PNIPAM-co-AAc gel were examined. The stress–strain curves were plotted with the average curves for each sample. Young’s modulus was obtained from the slope of the straight line in the linear region of the resultant stress–strain curves.

#### 4.5.2. SEM Analysis

SEM observed the morphological characteristics of the freeze-dried PNIPAM-co-AAc hydrogels. Before analysis, the hydrogels were swollen and equilibrated in distilled water at room temperature. They were rapidly frozen and then freeze-dried for 2 h in an freeze dryer (FDU-1200; Tokyo Rikakikai Co., Ltd., Tokyo, Japan) under vacuum at −65 °C. SEM imaging was performed on a Hitachi High-Tech S-3400N with an accelerating voltage of 15 kV. High-resolution images were obtained to elucidate the hydrogels’ surface topography and porous structure.

#### 4.5.3. FT-IR

Dried hydrogels were pressed into disks for FT-IR studies. FT-IR spectra were obtained by a transmission method using a Vertex 70 (Bruker Optics, Ettlingen, rmany) over the spectral range 1000–4000 cm^−1^. Spectra were recorded by averaging 50 scans with a resolution of 4 cm^−1^.

#### 4.5.4. Temperature-Swelling Ratio Phase Diagram

Hydrogel disks of 10 mm diameter and 0.5 mm thickness were immersed in ultrapure water, placed in the slot of a 1 mm deep glass bottom dish, and covered with a transparent plastic film to prevent air from entering. The dish containing this gel disk was placed on a temperature-controlled glass window cell (BH-302, Yamato Scientific Co., Ltd., Tokyo, Japan) and incubated at 20 °C for 1 day. After the incubation, the bath temperature was meticulously controlled with an accuracy of ±0.1 K and systematically varied between 20 °C and 40 °C at a rate of 0.02 K/min and returned. Visual observations of the hydrogel at the specified temperatures were recorded using a light microscope with a CCD camera (LPE-07W, Sanwa Supply, Tokyo, Japan). Simultaneously, the dish’s solution surface temperature was monitored using a thermographic camera (ARTCAM-320-THERMO-WOM-16, Artray, Tokyo, Japan). The hydrogels’ equilibrium swelling curves were obtained by evaluating the gel morphology and its area, indicated as S, using Image-Pro 6.0 software (Media Cybernetics, Silver Spring, MD, USA). The hydrogel’s swelling ratio at a given temperature *T* is expressed as *S*(*T*)/*S*_0_, where *S*(*T*) and *S*_0_ are the area of the hydrogel disk at temperature *T* and the equilibrium area at 20 °C, respectively. This process was repeated to construct the temperature-volume fraction phase diagram.

### 4.6. Observation and Analysis of Light-Induced Volume Phase Transition Behavior

Similar to the temperature-swelling ratio phase diagram experiment, the sample was left in a glass bottom dish at 20 °C for 1 day. While the dish remained static on the temperature-controlled glass window, the hydrogel disk was irradiated with light from an LED light source at an intensity of 1.62 × 10^3^ lm, covering a spot area of 1.5 mm in diameter. Visual documentation of the hydrogel’s dynamic behavior was obtained using the optical microscope with a CCD camera used in Section 4.5.4. Quantification of the irradiated gel areas was performed using Image-Pro 7.0 software, and gel sizes were determined by analogy with the method used for phase diagram delineation. The sample surface temperature was recorded periodically at one-minute intervals for thermographic analysis.

## Data Availability

The data presented in this study are openly available in article.

## Supplementary Materials

The following supporting information is available for download at: https://www.mdpi.com/article/10.3390/gels9110880/s1; videos: Volume change behavior after LED irradiation of PNIPAM-co-AAc gels prepared with bubble water left for (Video S1) 5 min, (Video S2) 30 min, (Video S3) 120 min, and with (Video S4) degassed water.
