# Supplementary material for: Additive-Free Method for Enhancing the Volume Phase Transition Rate in Light-Responsive Hydrogels: A Study of Micro-Nano Bubble Water on PNIPAM-co-AAc Hydrogels
Source: Gels. 2023 Nov 7;9(11):880. doi: 10.3390/gels9110880 (PMC10671373; doi:10.3390/gels9110880)
Supplement: Supplementary file 1 [file gels-09-00880-s001.zip › SI for bubble wate gel.pdf]

Additive-Free Method for Enhancing the Volume Phase Transition Rate in Light-Responsive Hydrogels: A Study of Micro-Nano Bubble Water on PNIPAM-co-AAc Hydrogels

Saho Kuroki, Masaya Kubota, Ryota Haraguchi, Yushi Oishi and Takayuki Narita *

Department of Chemistry and Applied Chemistry, Saga University, 1 Honjo, Saga 840-8502, Japan
* Correspondence: naritat@cc.saga-u.ac.jp; Tel.: +81-952-28-8805

Table of Contents

. MOVIE LEGENDS

Video S1: Volume change behavior before and after LED irradiation of PNIPAM-co-AAc gels prepared with bubble water left for 5 min. The hydrogel disk was placed on a glass bottom dish on a temperature-controlled cell (set at 20°C) and recorded using an optical microscope equipped with a video and CCD camera as the disk was irradiated with LEDs. The frame rate is 600x real time. The initial gel diameter in the movie is 10 mm.

Video S2: Volume change behavior before and after LED irradiation of PNIPAM-co-AAc gels prepared with bubble water left for 30 min. Conditions other than simple are the same as in Video S1.

Video S3: Volume change behavior before and after LED irradiation of PNIPAM-co-AAc gels prepared with bubble water left for 120 min. Conditions other than simple are the same as in Video S1.

Video S4: Volume change behavior before and after LED irradiation of PNIPAM-co-AAc gels prepared with the degassed water. Conditions other than simple are the same as in Video S1.
